# Real-Time Bibliometrics: Dimensions as a Resource for Analyzing Aspects of COVID-19

**DOI:** 10.3389/frma.2020.595299

**Published:** 2021-01-12

**Authors:** Daniel W Hook, Simon J Porter, Hélène Draux, Christian T Herzog

**Affiliations:** ^1^ Digital Science, London, United Kingdom; ^2^ Centre for Complexity Science, Imperial College London, London, United Kingdom; ^3^ Department of Physics, Washington University in St Louis, St Louis, MO, United States

**Keywords:** timescale, peer review, collaboration, gender, dimensions, COVID-19 crisis, full text search, real-time bibliometrics

## Abstract

Dimensions was built as a platform to allow stakeholders in the research community, including academic bibliometricians, to more easily create and understand the context of different types of research object through the linkages between these objects. Links between objects are created via persistent identifiers and machine learning techniques, while additional context is introduced via data enhancements such as per-object categorisations and person and institution disambiguation. While these features make analytical use cases accessible for end users, the COVID-19 crisis has highlighted a different set of needs to analyze trends in scholarship as they occur: Real-time bibliometrics. The combination of full-text search, daily data updates, a broad set of scholarly objects including pre-prints and a wider set of data fields for analysis, broadens opportunities for a different style of analysis. A subset of these emerging capabilities is discussed and three basic analyses are presented as illustrations of the potential for real-time bibliometrics.

## 1 Introduction

The COVID-19 crisis has changed the world on a grand scale. Its effects have been seen in every country, at every level and in every facet of life from social to professional. It is highly likely that the research landscape has been and will be fundamentally altered both in the short term and the long term as a result of COVID-19. The long-term issues are likely to include: funding for research; expectations regarding the public research sector's relationship with industry; expectations regarding the role of universities in sustainable development; and the role that institutions of higher education should be playing in retooling and up-skilling the workforce (Frey, 2019; Carden and Young, 2020; Hook, 2020; Hook et al., 2020; Wastl et al., 2020). While it is difficult to predict the future or even to guess the persistent long-term effects of COVID-19 on the research environment, COVID-19 does appear to have played the role of a catalyst and accelerant for change in the short term. We argue that the signal for some of these changes can already be observed in the data that is to be found in scholarly search databases and other modern technology-driven tools that support the research ecosystem.

In this paper, we propose the concept of “real-time” bibliometrics as a new capability for researchers, policymakers and analysts across the sector. The cornerstones of this emergent capability are: *data processing* that makes use of automated techniques (allowing timely data updates); and, *data delivery* via an API or other direct-access technologies (e.g., Google BigQuery) that give the user more scope to work with data directly without either the need to duplicate large portions of the database to derive insights, or the need for an expensive infrastructure. Tools that exhibit these types of approach include Microsoft's Academic Search, Allen Institute's Semantic Scholar, and Digital Science's *Dimensions*. Given the current authors' domain expertize, backgrounds and affiliation we have chosen to focus on Digital Science's *Dimensions* as the core for our analysis here. In addition, we have chosen specifically not to perform a product comparison as we feel that this would be better performed by others. Rather we illustrate the concept of real-time bibliometrics through three simple examples laid out below.

The idea of real-time bibliometrics suggested itself after we realized that there are four key features of the *Dimensions* platform that result from the original design aims, and which allow analysis of rapidly emerging events that impacts the research world, such as COVID-19. These are:

1. The inclusion of preprints and other content types such as awarded grants, patents, clinical trials, datasets, policy documents and scholarly (citation-based) and public (Altmetric) attention sources gives access to a broader range of potential signals for analysis^1^;
2. Inclusion of full-text search indices on all object types. Note that while all object types in *Dimensions* do have the capacity to have full-text associated with them for indexing, only 77 m of the 110 m scholarly articles are available for inclusion in the search index at the time of writing and that, for non-publications object, different bodies of text constitute full text (e.g., while for a patent this is the full text of the patent application, for a research grant this is typically limited to either a lay summary or a short abstract);
3. Daily data updates. It is a common theme that recently developed technology resources that support scholarship make use of machine-learning technologies. One way in which these technologies are deployed is to allow data enhancements such as subject categorization, person disambiguation and institution disambiguation to be applied in an automated way. This technology approach focuses curation resource on improving algorithms rather than improving individual data items. This shift in focus means that data can be added much more quickly to the Dimensions index and hence analysis can be performed daily;
4. The provision of programmatic/high-volume routes to access data. Many products now provide APIs that allow those familiar programming techniques to extract and analyze more data than is available in the web version of the product. Such APIs are demonstrated in the methodology behind the analyses included in the current paper. However, for even better real-time, high-volume access to data with the capacity to perform complex calculations in the cloud, mixing tools such as *Dimensions* with cloud compute platforms such as Google BigQuery, Snowflake or Amazon Redshift open up even greater potential. The *Dimensions* team have chosen to use the Google BigQuery platform to share their data. A free dataset that includes all COVID-19-related research objects from *Dimensions* can be found at (https://console.cloud.google.com/marketplace/product/digitalscience-public/covid-19-dataset-dimensions).

The combination of these facets allows analysts and researchers to carry out real-time bibliometric analysis. Historically, most bibliometric analyses do not require real-time data, however, we believe that the COVID-19 crisis has demonstrated one use case where this capability should be of broad interest and that this will lead to the development of further use cases where this style of analysis is relevant.

This paper does not attempt to be an exhaustive summary of all of the different use cases that may be explored in *Dimensions*. Here, we limit our attention to three examples related by their use of publication and citation data. Further examples that make use of clinical trials, grant and patent data may be found in Hook et al. (2020). Rather, the focus of this paper is on three basic analyses that do not make use of state-of-the-art bibliometric and scientometric techniques (such as those used in Suominen and Toivanen (2016); Zhang et al. (2016)), but instead focuses on simple approaches that demonstrate the potential of real-time bibliometrics in an easily accessible manner to a wider audience.

This paper is organized as follows: In the Methods section, we describe some of the key facets of the *Dimensions* database and the techniques that have been used to query the data. In the Results section, we have included the three analyses described above. Finally, we make some observations in the Discussion section. For brevity hereafter, we habitually contract COVID-19 to COVID.

## 2 Methods

### 2.1 Database

The *Dimensions* database comprises of a set of stores of data that hold information on different types of research inputs, research outputs (which we collectively refer to as “research objects”) together with the different types of attention accrued by those objects. The database constitutes a step towards a complete picture of the overall research landscape and helps to bring context to not only an individual piece of research, but also to a researcher, a research field, an institution, a funder, a country, and many of the other major research-related entities that may be of interest to stakeholders in the research world. *Dimensions* does this by merging openly available data with data from proprietary sources and enhancing both using persistent identifiers and technological approaches. Editorial guidelines for material to be included in the database are simple and transparent—there must be a reliable source for the data and each entity in the system must be associated with a recognized unique identifier. A more comprehensive description of how the database is constructed is included in Hook et al. (2018).

**FIGURE 1:**
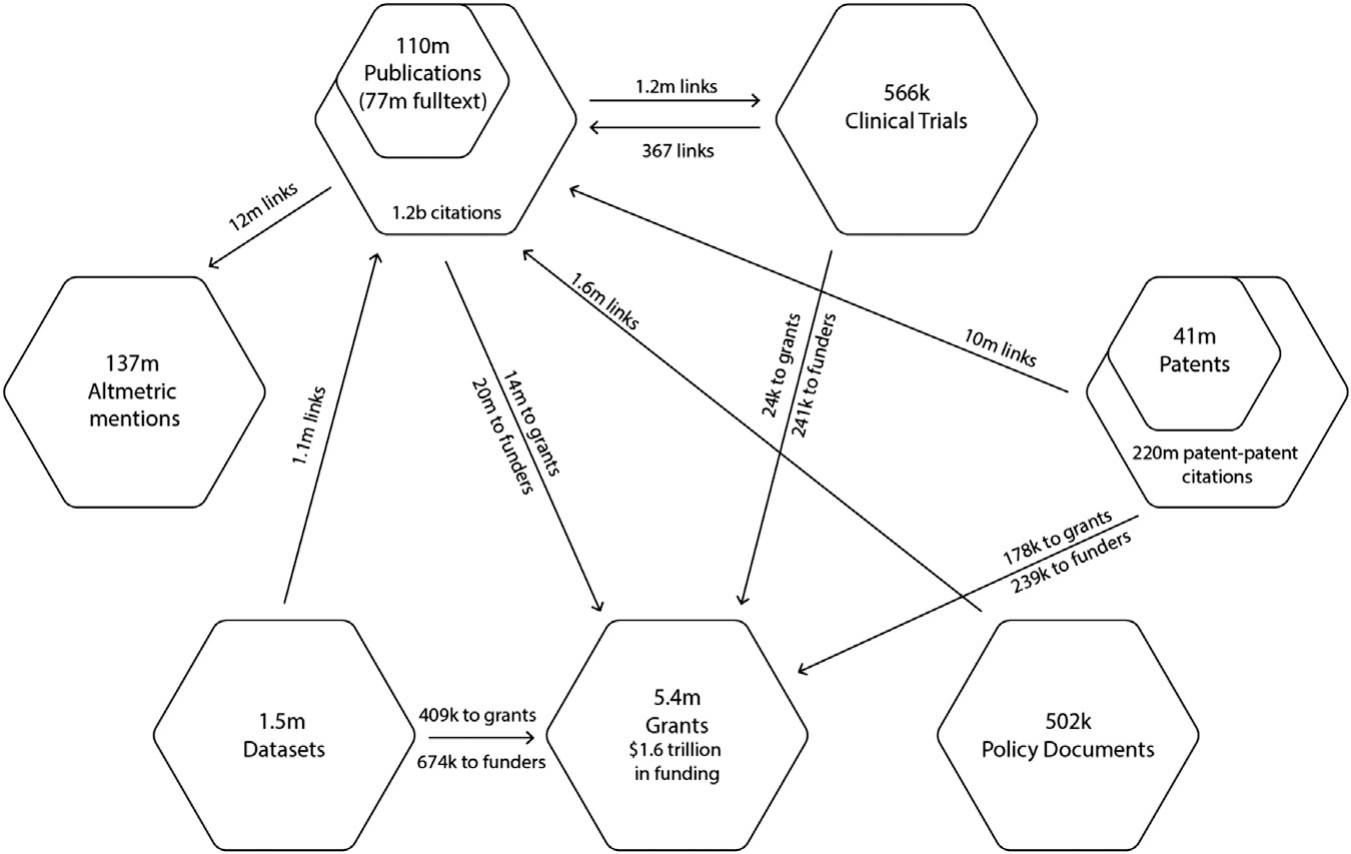
Volume of data holdings by object type and volume relationships between objects in *Dimensions* as of June 2020.

At the time that the analyses described in the following sections was performed, the *Dimensions* database contained more than 110 m publications (77 m with full text), 1.5 m datasets, 5.4 m grants, 41 m patents, 566 k clinical trials, 502 k policy documents and 137 m Altmetric mentions. Figure 1 summarizes *Dimensions*’ data holdings and the number of links between entity types at the time of writing.

To study the development of research related to COVID, we needed to construct a robust search string to identify material. Data Scientists in the Digital Science team collaborated with subject experts to formulate the Boolean query in the box below, which was used to search titles, abstracts and article full text, where it was available *in conjunction with a date restriction to 2020*.

“2019-nCoV” OR “COVID-19” OR “SARS-CoV-2” OR “HCoV-2019” OR “hcov” OR “NCOVID-19” OR “severe acute respiratory syndrome coronavirus 2” OR “severe acute respiratory syndrome corona virus 2” OR ((“coronavirus” OR “corona virus”) AND (Wuhan OR China OR novel))

This search string (together with the date restriction) was designed to be inclusive: by which we mean that it included all relevant outputs at the risk of introducing false positives. The definition of a false positive in this context is open to interpretation. For many, a false positive will be the addition of an article to the COVID dataset that is not centrally linked to COVID, but which only mentions COVID in passing without it being a central theme of the research output. Such articles *are* included in the results of this query. We deem this approach to be reasonable in the current setting since, in a broader sense, the inclusion of these non-central articles does provide a signal that represents of the level interest in the academic community relating COVID and helps to quantifying the overall level of research activity related to COVID. The articles included in this search results are also not limited to medical papers reporting infectious disease research, virology and vaccine-related technologies. The field of COVID-related research is significantly broader and includes not only research related to the disease and the effects of lockdown such as: epidemiology; public health; mental health and economics, but also, for example, socialogical issues such as the effect of the pandemic on minorities, the environment and tourism. The dataset that we find from *Dimensions* is also not limited to fully academic articles, but also includes academic news articles such as those found in Nature journal that have been given DOIs. Again, in attempting to quantify, classify and contextualize the output of the academy related to COVID, we do not see these articles as irrelevant to the present analysis.

For ease of access, the most current version of this query is linked to via the shortcut http://covid-19.dimensions.ai, which was recommended by the Chinese Academy of Science to help its researchers locate COVID-specific research (Kundu, 2020). The query was used to define further resources that have been made broadly available (for free and without the need for any subscription or data license): firstly, the resulting dataset is available on figshare (updated regularly) at https://doi.org/10.6084/m9.figshare.11961063.v21. There is also a live connection to the current data on Dimensions through Google BigQuery https://console.cloud.google.com/marketplace/product/digitalscience-public/covid-19-dataset-dimensions.

### 2.2 Data Extraction and Processing

All data featured the figures contain in the results section of this article can be found on Figshare (details in the data statement). Different types of data in *Dimensions* are updated with different frequencies. For example, per-article open access data is updated frequently in the source at Unpaywall, but *Dimensions* updates these data on a less frequent basis (every few days). Hence, while Open Access data in *Dimensions* is appropriately sourced to meet most use cases, it is not yet fully-aligned with the “real-time” biblioemtrics discussed here. As a result, when we perform the real-time analysis for this article, we actively query the Unpaywall database to ensure that the most recent data is included in the analysis.

Figures 4, 7, 8, 10, 11 are all produced using the *Dimensions* API. Figures 10, 11 use the Force Atlas 2 graph layout algorithm in the Gephi graph visualization software package (Jacomy et al., 2014).

**FIGURE 2:**
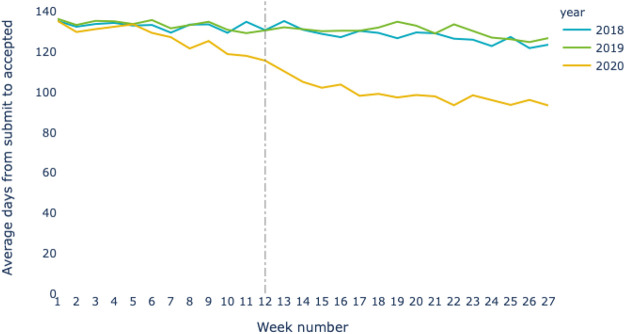
Average days between submission and accepted dates. The dotted line indicates week 12, mid-March 2020, where most of the world started entering in some form of lockdown, and schools were shutdown. For each week shown in the plot, the number of papers that have been accepted are taken and then amount of time taken since acceptance is calculated. The average time from submission to acceptance is then taken.

**FIGURE 3:**
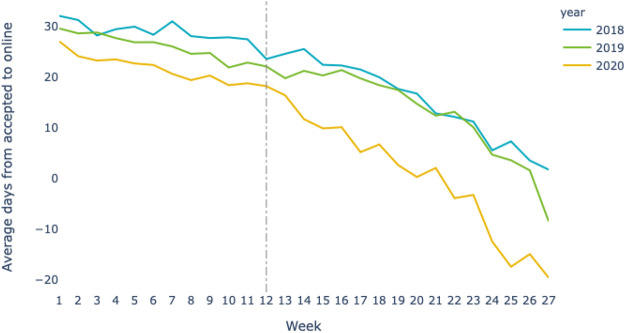
Average days between accepted and online dates. The dotted line indicates week 12, mid-March 2020, where most of the world started entering in some form of lockdown, and schools were shutdown. The value for each week is calculated by taking all papers in the Dimensions database that have appeared online in a specific week, calculating the difference between the date of online publication and the date of acceptance and then taking the average. When the average is negative, it indicates that the largest portion of articles in that week have been published online but not yet fully accepted (i.e. they are accepted with minor revisions).

Figures 2 and 3 are based on data that has been extracted from *Dimensions*’ full-text archive rather than the standard data that is made available in either the *Dimensions* web interface or API at the current time (although the data for this study are included in the data release vested on Figshare and associated with this paper). Around 20% of records in the *Dimensions* core have data for: date of submission, date of acceptance, and date available online and date of publication associated with them in a consistent manner that allows the analysis that we have carried out.

A further important aspect of the analysis behind Figures 2, 3 is that we can not rely on the COVID query defined above as the basis for a three-year comparison of behaviors. Since the query is defined and optimized to track the objects that relate to COVID-19, it does not pick up any results prior to 2020, and hence cannot be used to create a baseline for three comparable years. While we could perform a general research on “coronavirus” the number of research results from this field in previous years would lead to a dataset that would be too small for a robust statistical analysis. Instead, we argue that a robust proxy that includes much of the medicine-centric COVID-19 research today can be built by including articles in the following RCDC categories: “Infectious Diseases”, “Emerging Infectious Diseases”, “Clinical Research”, “Lung”, “Vaccine Related”, “Biodefense”, “Pneumonia & Influenza”, and “Pneumonia”. While this removes the non-medicine-related articles from the query that we have engaged with previously, it is these fields that are mostly likely to have had increased pressure to publish work and in which there is most likely to be a statistically significant effect. For each year in the study, papers accepted between 2nd January and 31st July are included. This choice of date is indicative of a specific class of data issue that we need to allow for in these analyses, namely the use of “default” dates in computer systems and metadata in scholarly publishing. In this case, that 1st January is used by many publishing systems as a “default” date used to represent not only 1st January but also, January as a month and the whole year. It is screened out in this analysis. We have included the first day in other months as, while these dates are also often used as a proxy for the whole month, this is a less statistically significant effect and leads to a proportionally lower error (up to 1 month) compared with up to 1 year in the January case.

Figure 12 makes use of a “gender guessing” algorithm that is applied to the author names associated with the articles and which classifies each author up to a certain tolerance using the first name of the author. We need to take a statistical approach given that this algorithm cannot achieve 100% accuracy, even if the data were perfect. Since we wish to use statistical approaches, the number of papers must be sufficiently large as for that style of analysis to be applicable. As a result, we considered publications across all subjects, regardless of their link with COVID research to form our baseline for this behavioral analysis. We used *Dimensions*’ data for the first six months of each of the last three years: 2018, 2019, and 2020. We calculated the proportion of women who had submitted articles every month, excluding authors whose genders were impossible to guess from their first name (i.e. names used for both genders or without enough information). We then calculated the difference in percentages for cooresponding months in each year.

## 3 Results

### 3.1 Timescales in COVID

The question of whether the system of scholarly communication is fit for purpose in the context of modern research is again being tested. The prodigious rise in COVID research has already caught the attention of many in the scientometric and scholarly communications communities (for example Brainard (2020); Colavizza et al. (2020); Torres-Salinas et al. (2020)) as well as the broader academic community. The emergence of COVID research as a new field, is taking place at a substantially accelerated rate compared to the usual development that one might expect in a usual situation. There are several caveats that must be drawn from the current situation. Firstly, that bibliometrics as a field is not well positioned with tools to support the analysis such a rapid expansion. Typically, bibliometricians and scientometricians are used to working on substantially longer timescales. Secondly, the definition of an emergent research area is seldom so cleanly and simply articulated as in the boxed search string above. The normal pace of development of a research field is usually inextricably linked to the speed of the emergence of technologies, theories, and discussion and socialization of ideas. However, in the case of COVID there is an powerful exogenous driver.

While it is tempting to think of COVID as a microcosm in which we can study the emergence of a field, with the parallel development of the social structures, in an accelerated manner, this is not the case. The development of the field is a development under stress and with a specific goal in mind for a large proportion of the field (a vaccine) and on a specific timescale (as soon as possible). We can define the core areas of COVID in general terms to be the search for a vaccine, the spread of the disease and the public health implications of the virus. A non-exhaustive set of adjacent areas might include: the study of the economic impact of COVID on global markets, the impact on specific sectors such as the travel industry, the economic recovery from COVID, the mental health aspects of an extended period in lockdown, the effects of the crisis on people based on race, social status, gender and age. In each case, advances in research in adjacent fields are often perceived to be under less time pressure than those in core areas related to health.

As a result, any analysis of the sociological behavior change of academia itself as a result of COVID, is likely to have limited applicability. It should be thought of as a case study of a system under stress and consequently lessons that are drawn from such an analysis are relevant to comparable systems and should not be considered to be generally applied.

Nonetheless, we are observing elements of culture change during this period that may survive the immediate crisis. The current stressful situation is also highlighting several deficiencies in the structure of scholarly communications and a variety of social issues in academia at large (Minello, 2020; Viglione, 2020). In this section, we will specifically, study the need to publish at speed in the current situation, the format of publication, verification of results, and access to those results.

The first analysis presented in this paper concerns the change in publication practices in the community as a result of COVID. During a period of epidemic (or indeed pandemic), the work of the research community becomes more time-critical. The speed with which results are shared between researchers is a key factor in developing approaches to saving lives. International barriers, considerations of professional academic advancement and frameworks of evaluation take second place to solutions that improve the chances of survival of those infected. In the case of COVID, a vast number of researchers from around the globe have turned their interests to COVID research (Hook et al., 2020), with the effect that the volume of publication in this newly emergent area has reached more than 105,000 publications in 6 months - this constitutes around 3% of the world's research output so far this year. As a matter of comparison, other fast moving areas such as “Deep Learning”, have taken more than 6 years to reach a comparable number of outputs in total. In 2019, Deep Learning achieved a total of 99,000 publications in a single year, following a decade of development. A number that COVID eclipsed after just 6 months in 2020.

The need to share advances more quickly has led to two related developments: i) the use of open access to ensure that all developments are shared globally, and ii) the use of faster publication routes. Both of these needs are met by the preprint publication format as preprints are both open access and, since they pass through no peer review process, they are instantly available to the community. The lack of peer review in preprints has given rise to a wide range of concerns (Kwon, 2020). Academic publishers have been quick to engage with these challenges firstly by making COVID-related publications available in through open access channels and secondly by decreasing peer review times.

Preprint publications have minimal submisssion-to-publication times and have become well established in some fields as a way not only to rapidly communicate research results but also to establish priority. There are, however, well documented challenges with low-touch or no-touch review (Chiarelli et al., 2019). Many peer review servers have a short delay between submission and publication in order to do basic checks on manuscripts. However, this delay is typically on the order of a few days. By comparison with the traditional process, the average time from submission to acceptance and on to online publication of a manuscript, averages around 170 days. Figure 2 shows the average time from submission to acceptance based on the availability of data submission and acceptance dates in the full text records of the *Dimensions* databsae for the subset of fields involved in COVID research as discussed in Section 2.2; Figure 3 shows the average time from acceptance to online availability on the same basis as the previous plot. In each plot data is shown for the three years 2018, 2019 and 2020. The 2018 and 2019 lines establish the average time of the state-of-the-art in either peer review or post-peer review manuscript processing. In Figure 7 the time from submission to acceptance is fairly constant at 130 days whereas, in 2020 (yellow line), this time has reduced by around 40 days to less than 100 days. In Figure 3 we see that the average number of days from manuscript acceptance to online availability is generally trending down. This, we speculate, is a result of improvements in publishing processes and increased willingness of publishers to post manuscripts that are accepted subject to minor changes or which are already part of a preprint to review pipeline. However, the 2020 line (yellow) in this figure shows that this part of the process also has significantly decreased during the COVID period.

**FIGURE 4:**
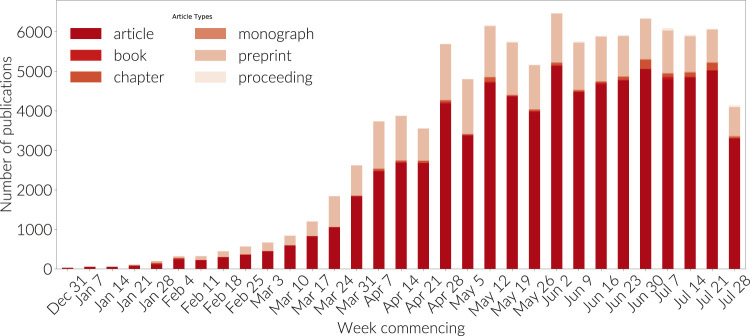
Research output results from querying with the boxed COVID search definition in Dimensions. Outputs are grouped week by week and are not shown cumulatively. Output types as per the legend.

**FIGURE 5:**
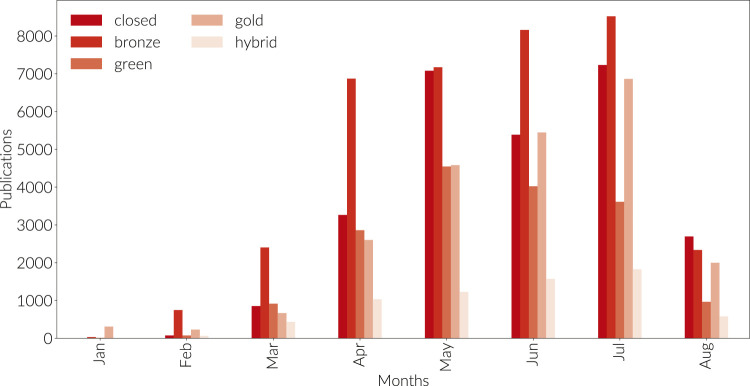
Open Access research output resulting from querying with the boxed COVID search definition in Dimensions. Outputs are grouped month by month and are not shown cumulatively. Output types as per the legend.

Peer review forms a critical piece of the scholarly communication process, ensuring the validity of research before it is broadly shared. However, it is a slow process as the comments of peer reviewers are addressed and responses iterated between authors and reviewers. The typical periods of peer review were clearly long enough during the early era of the COVID crisis to induce researchers to try out preprints as a mechanism to share their research, as seen in Figure 4. Hook and Porter (2020) noted that preprints have rapidly become established as a mainstream research output. Several other analyses have also appeared to examine this phenomenon Fraser et al. (2020). We speculate that preprints have not gained more traction in medicine in spite of COVID as a motivating influence due to a combination of effects:

1. substantial progress toward finding a vaccine may have alleviated pressure to share results rapidly;
2. Increase in speed and efficiency in the speed of peer review, as demonstrated in Figures 2, 3 and discussed by (Eisen et al., 2020; OASPA, 2020);
3. publisher commitments to make COVID-related papers available through Bronze Open Access (as illustrated in Figure 5), as well as early publication of manuscripts that have completed the peer review process but while they are still in production Carr (2020a, b); Kiley (2020);
4. concerns over circumvention of the peer review process as a quality check (Chiarelli et al., 2019; Johansson and Saderi, 2020; Kwon, 2020).

In summary, even in spite of the need for speed of communication, there is a competing need to ensure that information can be trusted and hence researchers continue to need access to infrastructures that allow for the trustful dissemination of research both within and without the community.

### 3.2 Evolution in Collaboration

It is seldom that it is possible to examine the emergence of a field in real time. In the case of COVID research, we have an unparalleled opportunity to do exactly that. However, care needs to be a taken, this is not the typical growth of a field. To borrow a concept from physics: when crystals grow in a natural environment they have a certain structure and uniformity; however, when they are fabricated in conditions that accelerate their growth, there are often different features and defects that emerge, and so it appears to be with the field currently establishing around COVID research. The field is drawing from many other specialisms and is accessing different networks in different geographies. Initially, development of research followed the incidence of the outbreak of the disease - starting in China, moving to Europe and eventually to the United States. This spread is reflected both in publication and clinical trial activity (Hook et al., 2020).

Through the data in *Dimensions* coupled with full-text searching capabilities it is possible to see this evolution day-by-day, week-by-week. In this section of the paper, we relate a high-level analysis of the development of the field of COVID research and the development of the international picture of collaboration during this period of development.

**FIGURE 6:**
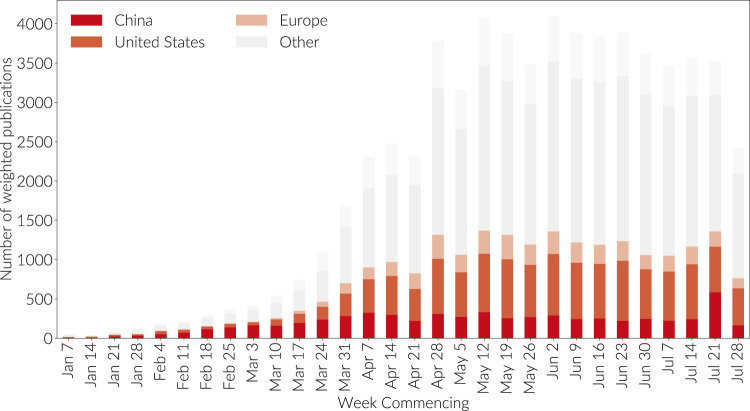
Weighted publication counts attributed by country in proportion to researcher affiliation. This graph is not cumulative. Papers are binned by the week commencing on the dates given; the final week is incomplete.

First of all, we look at the rise of COVID publication by country so that we understand how the geographic locus of COVID research has developed with time. Figure 6 shows the level of publication production, highlighting the top producing countries. It is generated using the GRID database of institutional affiliations (see http://grid.ac). For each publication where there are institutions associated with the authors, the paper can be partitioned into the contributions from each country. A normalization is applied such that each paper continues to contribute a count of one in total across all contributing countries. Hence, if a paper is co-authored with two authors associated with institutions in the US and three authors associated with institutions in China, then 3/5 of the paper will be attributed to China and 2/5 of the paper to US. The graph is not cumulative but rather it represents the number of papers appearing in the week commencing at the date marked on the axis. The top 12 producing countries (over the full time period) are listed explicitly, countries outside the top 12 producers in aggregate over the period are agglomerated into “other”, authors (proportions of the paper) associated with institutions that contributed but which are unknown to GRID or which cannot trivially be mapped to GRID are listed as “No Afiliation”.

**FIGURE 7:**
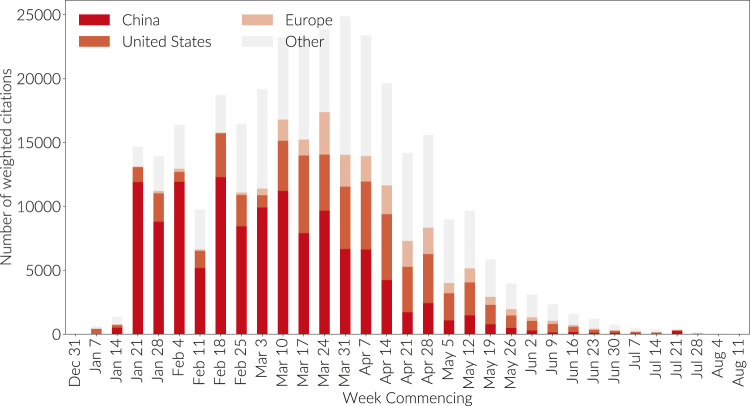
Fractional citations to all COVID publications by receiving country determined by researcher affiliation. Totals by week in which citations were made. COVID research is defined relative to the boxed query in this report. Only citations from other COVID papers have been considered. All numbers are derived from Dimensions on May 24, 2020.

From Figure 6 we see, unsurprisingly given the earlier need for a vaccine and (unfortunately) the availability of infected subjects, that China took a leading position in the early development of COVID research. Since early April, however, China has plateaued in research volume and the main growth has been in the US and European research base. However, China's first-mover advantage established its publications as foundational to this new field in both in highly respected journals and in shear volume of citations. Figure 7 Shows the COVID-related citations made in each week by country of target publication. Hence, if a new publication was published in the first week of May citing a paper from February on which 50% of the authors were affiliated with Chinese institutions and 50% with United States institutions, then a value of 0.5 will count toward the orange color representing the United States bar in the May 4 bar, and 0.5 will count toward the deep red bar representing China. It is clear that Chinese research has receive a great deal of attention.

Table 1 lists journals ordered by the number of COVID-related citations they have received. A COVID-related citation is defined to be a citation to an article that is returned from *Dimensions* in response to the boxed query. The “No. of Pubs” column lists the number of COVID research outputs published by the venue until May 24, 2020—note the high volumes for the preprint sites medRxiv, bioRxiv, and SSRN. The paper totals in the table are not rounded fractional counts but whole papers that involve either a US-based, China-based or EU-based author respectively–hence, there will be double counting between the US, CN and EU columns in the case of collaborative research. Our own analysis (below) shows significant collaboration within established international networks, albeit at a low rate relative to “normal running”. This analysis is supported by the results of Fry et al. (2020). The EU is defined to include the EU-27 countries, the United Kingdom, Norway and Switzerland.

**TABLE 1:** Regional representation of COVID research by publication venue. Publication venues include journals and preprint servers and are selected (and ordered) by number of citations to COVID articles (Total Cites column). COVID research is defined relative to the boxed query in this report. All numbers are derived from *Dimensions* on May 24, 2020. Note that arXiv.org contains 1,013 publications related to COVID, however, the quality of address metadata and citation details of these papers in *Dimensions* does not currently allow it to be included in this analysis.

Journal	No. of pubs	Total cites	No. of pubs			Citations		
			US	CN	EU	US	CN	EU
The lancet	188	11,643	64	41	115	1,232	9,614	2,410
New England journal of medicine	181	10,599	114	20	35	3,267	4,496	1,101
JAMA	136	6,781	108	13	10	1,618	4,305	588
medRxiv	2,867	4,431	994	765	1,010	1725	2,423	1,560
Journal of medical virology	271	2,983	50	148	51	460	2,543	334
bioRxiv	880	2,455	391	189	291	802	1,397	616
Radiology	52	2,370	18	20	12	463	1897	52
Nature	22	2,247	9	13	7	119	1957	212
The lancet infectious diseases	114	2,161	32	34	53	524	1,139	828
The lancet respiratory medicine	58	1971	21	13	34	270	1,479	431
Clinical infectious diseases	136	1,661	51	59	31	297	1,359	200
Science	76	1,536	43	24	25	1,072	890	671
International journal of infectious diseases	128	1,374	27	68	34	635	1,093	433
Eurosurveillance	64	1,328	4	9	55	177	495	1,018
The BMJ	399	1,279	38	12	339	74	383	775
International journal of antimicrobial agents	40	1,041	6	11	14	0	110	667
Cell	18	1,005	8	5	9	276	70	929
Emerging microbes & infections	43	846	9	35	3	91	805	8
Journal of infection	146	840	8	103	39	25	788	116
SSRN Electronic journal	1,655	705	576	436	520	316	354	142

**FIGURE 8:**
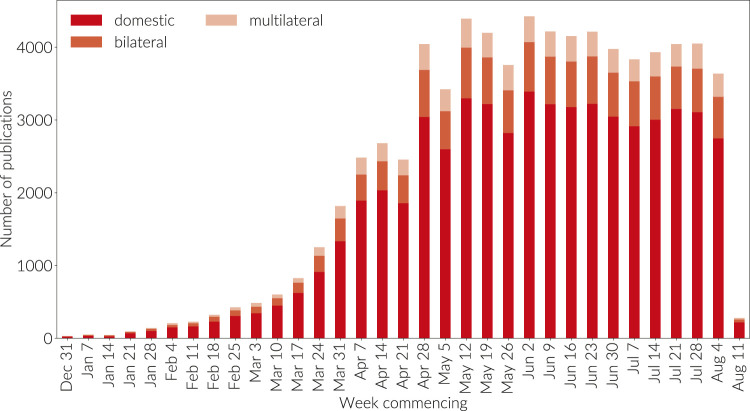
Count of domestic, bilateral, and multilateral country collaborations. This graph is not cumulative. Papers are grouped by the week commencing on the dates given; the final week is incomplete.

The international multidisciplinary science journal Nature asserted that politicians can learn from researchers’ collaboration habits (Skipper, 2020), but while we see strong collaborations on the scaffolding of established academic networks (Fry et al., 2020), it is clear from our analysis that the overall proportion of bilateral (specifically two countries) and multilateral (more than two countries) research collaborations is still embryonic. Indeed, Figure 8 shows that while the proportion of internationally co-authored work is steady, the vast majority of research on COVID to date has been authored within countries. It is well established that international collaboration is rising across subjects (Adams, 2013) so we interpret this graph to show the early stage of the field.

**FIGURE 9:**
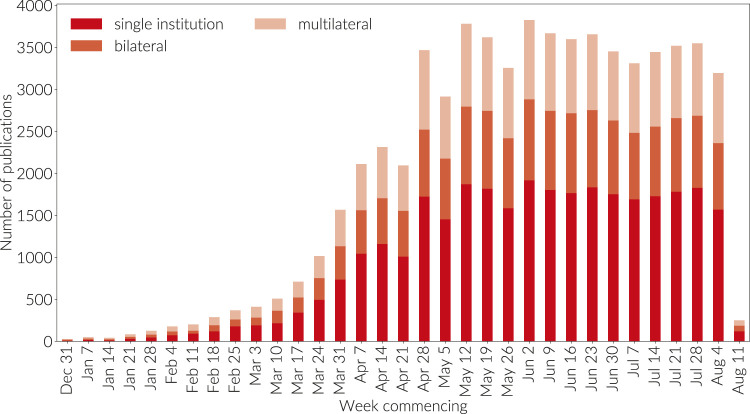
Institutional collaboration modes for COVID research. This graph is not cumulative. Papers are grouped by the week commencing on the dates given; the final week is incomplete. Publications counting toward ‘single institution’ have only a single institutional affiliation, bilateral publications are affiliated with two institutions, and multilateral publications are those that are affiliated with more than two institutions. There is no sensitivity in this plot to the country in which institutions are situated.

There are several factors beyond the nascent stage of COVID research that may have contributed to early trends in international collaboration. Firstly, China is a strong contributor to the data in the early months of Figure 8. China's research capacity has been growing so rapidly that the rest of the world lacks the capacity to keep up with China's expanding research base and hence, despite becoming the favored collaboration partner with a growing number of countries around the world, the international footprint–the ratio of domestic to international papers in China–is currently against the world trend. The international picture is mirrored at institutional level as can be observed in Figure 9. During January, February and March a significant proportion of research took place not just within a single-country setting but also within a single institution setting. While this remained the dominant behavior in April and May at the country level, we can see the emergence of greater inter-institutional collaboration in these months as the collaborative network starts to establish and a stabilization of inter-institutional and international collaboration at more normal levels in June, July and August.

A large proportion of the research in Figure 8 is medical. Hence, we may speculate that a further potential effect at play in Figure 8 is that many researchers may feel pressure to make headway with a vaccine. As a result they are, in the early period of their research, focusing on developing their understanding of COVID rather than developing international collaborations. This tendency may be compounded by the nature of funding that is emerging in many countries, which is small scale, and targeted at small groups or individuals. This may make sense since the complexity of developing a COVID vaccine was, in the early period of the research, not well understood. It appears simply to take time to establish relationships on a new research topic, even when connections are already in existence.

Each of the two figures has the same basic structure, but different coloring has been applied to emphasize different aspects. Each of these figures depicts 50,979 researchers, each of whom has published a COVID paper. These researchers are derived from the *Dimensions* person graph and hence are not dependent on address information from COVID papers to derive these visualisations. The 488,188 researcher-researcher links represented in the diagram are not identical (i.e., links between co-authors are not duplicated with multiple co-authored papers), and relate to any relationship that has been established through the whole research career of the researchers involved, not only the COVID period of research. Thus, these figures show the full “COVID-activated” network of researchers.

**FIGURE 10:**
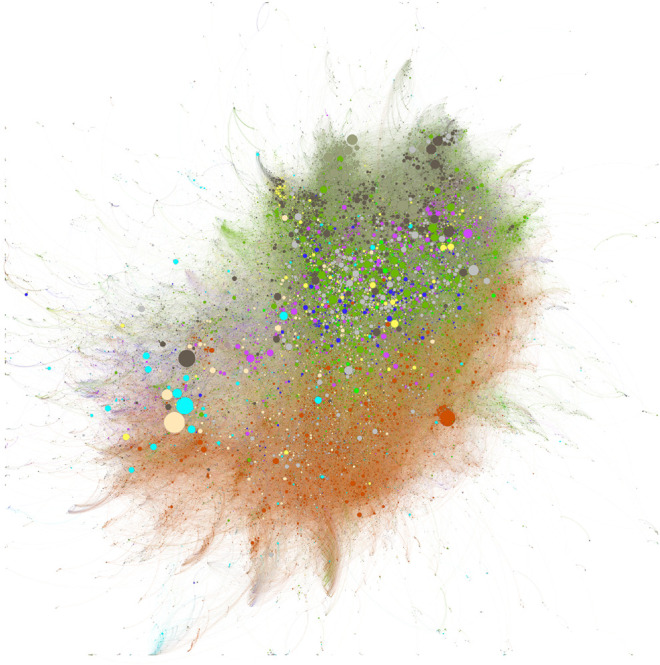
Research Collaboration among COVID Researchers. Researchers colored by primary RCRC category. Clinical Research (green), Infectious Diseases (orange), Cancer (dark brown), Genetics (light brown), Cardiovascular (olive), Lung (dark blue), Digestive Diseases (purple), Neurosciences (yellow), and Bioengineering (light blue). Clustering is based on proximity of co-authorship. Node size is determined by number of publications in whole research career.

In Figure 10 the confused distribution of colors makes it clear that COVID is already highly interdisciplinary with respect to the NIH's RCDC categorization scheme, which classifies different disease areas. Broadly, three areas emerge: first, the area characterized by the mix of cardiovascular (olive), clinical (green), lung (dark blue), neurosciences (yellow) and digestive diseases (purple); second, an area to the south of this highly mixed patch that is dominated by infectious diseases (orange); lastly, the peripheral group on left of the figure with a prevalence of light clustering of bioengineering (light blue) and genetics (light brown). This complex landscape indicates how multifaceted this research area has already become. Under this categorization, neither preventive medicine nor epidemiology/public health, both mainstays of the overall body of research in this area, emerge as coherent collaborative blocks.

**FIGURE 11:**
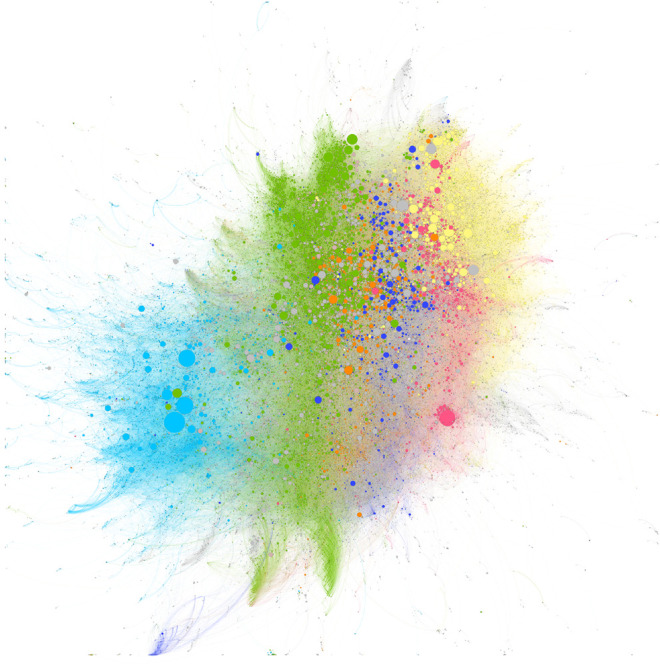
As Figure 10 but with researchers colored by country of current affiliated institution. China (light blue), US (green), United Kingdom (orange), Germany (dark blue), France (pink), and Italy (yellow).

Figure 11 shows the same background as Figure 10 but is colored by the current country of the institutional affiliation of each researcher. It is clear from this version of the graph that the clustering, and hence the overall structure of the network, is much more influenced by geographic collaborations than by subject collaborations. This is entirely in line with our findings from Figure 8, where we saw a high percentage of domestic collaboration and Figure 9 where we even saw that institution-specific localization was still significant at this time. We see distinct ‘banded’ collaborative structures for each of the main COVID-researching countries: China (light blue) on the left, collaborating most strongly with the US (green), which is highly integrated with the United Kingdom (orange) and Germany (dark blue), which are, in turn, integrated with France (pink) and Italy (yellow). The European countries show a high degree of integration, with the United Kingdom being highly collaborative and hence more diffuse in the picture.

Both Figure 10 and Figure 11 are subtle to interpret. However, one way to think of this network is as follows. All the researchers represented in the plot have published a COVID paper. Since we have clustered them based on their prior collaboration history as well as the COVID collaborations, we can think of each link as having a particular state of color: If a collaboration between two researchers does not contain a co-authored COVID publication, then we could color the link grey, and if it does contain a COVID publication, then we could color the link red. To assess how much of the collaboration graph has been accessed/created as a result of COVID, we can look at the proportion of the graph with grey links vs. red links. In this case, we would find that 57% of the connections are COVID related (which would drop to 45% if we considered only established researchers). Hence, COVID has lead to significant new collaborations, while at the same time accessing a large proportion of the existing collaboration network.

Ironically, this is precisely the type of thinking that disease modellers and epidemiologists would use in agent-based models to study the spread of a disease. In this case, the disease would be ‘doing research into COVID’, exposure would start with reading something in the media or in the research literature, infection would be starting research, and recovery would be publishing a paper. Indeed, understanding the sociology of research that is emerging from the COVID microcosm might well benefit from disease modelling techniques.

### 3.3 Gender Imbalances

In this final section of the analysis, we look at how typical gender roles have impacted the sociology of scholarly communication during the emergence of the COVID period. Recently, this topic has come to the forefront of the scholarly communication discussion (Donald, 2020; Matthews, 2020; Minello, 2020) In the analysis that follows, we have chosen to use first co-authorship as a means by which to benchmark the level of gender bias in the research environment. Of course, it can only be a proxy and cannot possibly give a complete picture. In addition, there are many fields that publish co-author names alphabetically and our analysis would not be valid in those fields. However, rather than attempt to quantify a very uneven landscape, we have assumed that alphabetic ordering is either in a minority or does not overly skew our results.

**FIGURE 12:**
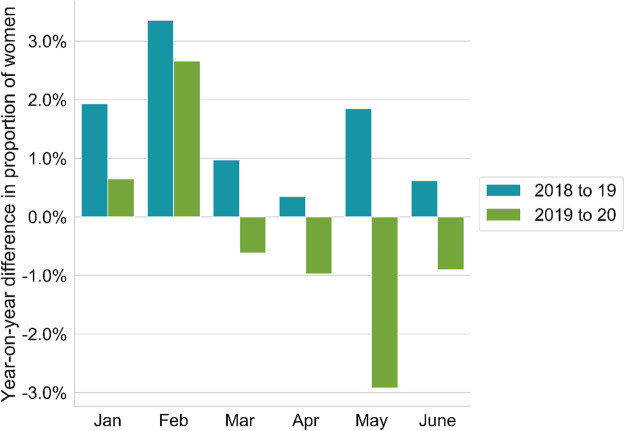
Annual comparison of the progression of female first-position co-authorships on global output. The bar chart shows the percentage change in the number of women appearing as co-authors on papers in global output. Gender of co-author is determined using a gender guessing algorithm. A co-author is classified as a woman in the case that the algorithm indicates a confidence level of more than 80%. Note that this plot is not limited to COVID-related publications and is carried out on all publications with a publication date in the month in question. The blue bars show the positive trend toward a greater number of female first author papers from 2018 to 2019. The green bars show the less favourable trend from 2019 to 2020 as the increase in the number of female first-authors at first slowed in January and Febrary 2020 and then declined at an increasing rate in March, April and May.

Figure 12 summarizes the impact on the equality of gender represented in publications during the opening months of 2020.

Again, for this analysis, the ability of *Dimensions* to supply timely data that allows a real-time month-by-month analysis is insightful in drawing conclusions regarding sociological issues in research. In this case, the result is marked. During the COVID period, lockdowns have been implemented around the globe. COVID lockdowns have become a target of significant academic study in their own right: the mental health implications, as well as the impact on different countries and different social strata are not only interesting from an academic standpoint but critical to inform policy. Closer to academic research itself, we can see an immediate manifestation of lockdown emerging clearly in Figure 12.

Throughout the first three months of this year, countries entered a period of lockdown. In the case of China, lockdown started to be introduced in February. By the end of March, a significant proportion of China, Japan, much of Europe, the United Kingdom, and the US were all in lockdown. Effects of the lockdown included researchers being unable to access their facilities in their institutions (unless they were directly work on vaccine-related COVID research). It also lead to the closure of schools. Many researchers took the opportunity to write up and complete previous papers, leading to an overall rise in the papers received by publishers. More than 90% of researchers claimed that COVID had impacted their ability to conduct their research to some extent and that they would need to rely on existing data that they had already harvested from their experiments to continue their work (Nature, 2020). However, the burden of the closure of schools appears to have fallen disproportionately on women. Figure 12 shows a clear signal for this conclusion as the number of female first authors decreased markedly just following the beginning of the most extensive phase of lockdown in March. An effect that we see gradually reducing as schools gradually reopened in June or a “new normal” began to emerge. We speculate that this decrease in female first authorships is a direct result of the closure of schools around the world. The main body of the world's research continues to be produced in the US, China, United Kingdom, Germany, France, Italy and Spain. These were all countries that were heavily affected by COVID and which imposed lockdowns that included school closure. They are all societies in which there is a tradition of females having the principal responsibility for raising children. While this trope has been hidden to some extent by progressive policy choices, there is clearly a continuing imbalance that emerges from the data in the simple analysis presented here.

## 4 Discussion

As with any analysis that has been carried out at the time at which a sociological effect is developing, the insights shared in this paper are very much of the period in which they were generated. They lack the “wide-angle lens” of history or the much more considered analyses that will come. The analyses contained in this paper are also specifically not designed to be cutting-edge bibliometrics analyses, and this would then focus the reader on the nature of the analyses rather than the capabilities of the data source that is powering those analyses.

Dimensions and other resources that follow similar approaches focus on providing a data to empower researchers and analysts to ask questions and then to use technology to move more quickly to analysis and interpretation rather than on gaining access to data. In previous work, we have described the nature of the *Dimensions* data system, focusing on the interlinking of different data types, data enhancements, and the use of persistent identifiers. We have also spoken about the ethos of building the system and the values behind it Hook et al. (2018); Herzog et al. (2020). However, the COVID-19 crisis has highlighted a combination of features of *Dimensions* that suggest a new style of analysis is not only possible but potentially valuable, and which may even be required to better support objective decision making in an era of increased uncertainty.

Many bibliometric analyses have the advantage of time: that distance from an event that provides the ability to take a longer view. This is well-matched to an analysis that can consider data from years rather than months or weeks. Yet, in the face of the COVID-19 crisis is it precisely an analysis over months and weeks that is required to be able to track trends and to prepare well-informed policy and responses to policy. We refer to this approach to analysis as “real-time” bibliometrics. While it is clear that this approach has significant limitations in the perspective it can bring, we believe that is can also be seen as a useful tool in helping to pinpoint, quantify and respond to trends as they happen, for all stakeholders in the research ecosystem.

The enablers of real-time bibliometrics that we have observed from doing the analyses contained in this study are: i) the use of technology that can allow data to be extracted and enhanced without the need for human curation of individual records (rather human curation should focus on activities that improve inputs to algorithms); ii) swift updates to data enabled by i); iii) the automated application of categorization at a per-object level; iv) an inclusive approach that makes a broad range of data types and fields available for study; v) full-text searching that allows maximal freedom to explore data; vi) technologies that facilitate programmatic access and manipulation of data. This is not an exhaustive list of features, but these are the ones that emerge from the analysis performed both in the current paper and in (Hook et al., 2020).

It remains unclear whether real-time bibliometrics is something that is either valuable in a general context or, indeed, would be an advisable route from either bibliometricians or scientometricians to follow. One should clearly be cautious before deploying such technological approaches as these in a policy environment since the perception of an active measurement or overly active feedback mechanism can have negative social effects or drive unwanted behaviors Goodhart (1981); Strathern (1997). However, the current authors believe that developing a greater understanding of the potential for real-time analyses and their potential impacts should be of broad academic interest and may eventually find a role in policy formulation. Defining “real-time bibliometrics” in a more rigorous and well-structured manner, broadening the range of tools that are available to researchers and analysts, as well as identifying pitfalls, challenges and undesirable effects of this style of analysis would be of general value.

## Data Availability Statement

The datasets presented in this study can be found in online repositories. The names of the repositories and accession numbers can be found below: 10.6084/m9.figshare.c.5092994.

## Author Contributions

Ideas for this article were generated and refined by DH, SP, and CH. Data analysis for this article was performed by SP and HD. Interpretation of analysis was performed by all co-authors. The article was drafted by DH and all authors collaborated on editing the article and responding to referee comments.

## Conflict of Interest

All co-authors of this paper are employees of Digital Science, the creator and provider of *Dimensions*.

## Footnotes

^1^ While analyses of all of these different object types is not included in the current paper, further examples may be found in (Hook et al., 2020).
